# A New Alternative Surgical Treatment of Hallux Valgus, in Moderate to Severe Cases of the Disease With a Two-and-a-Half-Year Follow-Up

**DOI:** 10.7759/cureus.14334

**Published:** 2021-04-06

**Authors:** Prodromos Natsaridis, Vaios Goulas, Themistoklis Poulios, Vasileios Akrivos, Christos Alexandropoulos, Stefanos Tsourvakas, Aristeidis H Zibis

**Affiliations:** 1 Orthopedic Surgery, General Hospital of Trikala, Trikala, GRC; 2 Orthopedic Surgery, General Hospital of Volos, Volos, GRC; 3 Anatomy, School of Health Sciences, University of Thessaly, Larissa, GRC

**Keywords:** halux valgus, chevron, osteotomy, modified, mcbride, cannulated screw

## Abstract

The study aims to evaluate the treatment of moderate to severe forms of hallux valgus with the lowest invasiveness in soft tissues and especially with an alternative modified Chevron osteotomy of the first metatarsal. Additionally, it emphasizes the necessity of the modified McBride procedure (capsuloplasty and release of specific concrete soft tissue structures) and the importance of the soft tissue manipulation in the particular surgery intra-operatively, as well as postoperative medical and personal care and duration of rehabilitation. Patients with an average age 58 years (range 51-65), who underwent a Chevron type osteotomy with combination of soft tissues interventions laterally and medially of the first metatarsophalangeal joint, for symptoms they had of systematic hallux valgus without any other degenerative problems in metatarsophalangeal joint between 2017 to the beginning of 2018, were retrospectively reviewed with an average follow-up of 29 months (range 26-31).

## Introduction

A large patient population suffers from hallux valgus more commonly women [1], with the disease appearing, in many cases, from young age. The surgical treatment of the disease with the appropriate surgical technique when surgery is indicated for correction of the deformity (with hallux valgus angle and intermetatarsal angle less than 36° and 17°, respectively) is controversial, as there is a high rate of recurrence of the deformity, often a short while after the surgery. One of these procedures is the classical distal first metatarsal Austin/Chevron osteotomy, a relatively stable V-shaped osteotomy that is commonly used for mild to moderate deformities.

The combination of interventions like a modification of the Chevron osteotomy, also probably for some surgeons the more distal to the first row of the foot-phalanx osteotomies (Akin osteotomy) and a modification of soft tissues intervention (release of Hallucis adductor & capsuloplasty) allow corrections of larger than moderate deformities. There are also other types of osteotomies including proximal metatarsal dome osteotomy, Lapidus and Scarf but these techniques were not used in this study separately or even in combination with an other technique. Almost all of the varied techniques associated with Chevron osteotomy are performed with two straight oblique osteotomies to the long (horizontal) axis of the first metatarsal, limiting the possibility of correction of larger deformities as they disrupt the stability of the joint and because there is often a lack of the appropriate instrumentation to perform them. Additionally, even in simple to moderate deformities, in which the classical Chevron osteotomy can be performed, the fact that there is excessive tissue damage, a poor choice of surgical techniques and materials for restoration, as well as a lack of respect for the anatomy of the region and the soft tissues, all induce complications that repel the patients and the surgeons from the surgical treatment of the deformity.

An innovative, alternative technique is now proposed based on the classical Chevron osteotomy, which involves a change in the angle and the directions of the two osteotomies (dorsal and plantar osteotomies), keeping the dorsal osteotomy almost vertical to the longitudinal axis of the first metatarsal and changing simply the angle of the plantar osteotomy according to the degree of the deformity and the desirable correction. Thus, it increased the endogenous stability and secured the effectiveness of the osteotomy, having a larger cut bone surface and amplifying the stability with a cannulated compression screw.

## Materials and methods

Materials

This was a retrospective - with prospectively collected data - study of patients in a two-year period from October 2017 to May 2018, a total of 36 patients were enrolled in this cohort study, five (13.9%) men and 31 (86.1%) women. The average age of the patients was 58 years (ranging from 51 to 65 years). The follow-up period ranged from 26 to 31 months. Inclusion criteria were difficulties and foot deformities. Exclusion criteria were metatraumatic hallux valgus, revision treatment hallux valgus, any type of foot infection or other paralytic or mid-foot or hind-foot deformities. All patients underwent surgery (modified Chevron osteotomy and modified McBride procedure), mostly due to discomfort and intense regional and metatarsal pain. All operations were performed by the same Orthopaedic surgeon (P.N.). All the patients were clinically assessed using the American Orthopedic Foot and Ankle Society (AOFAS) and hallux MetaTarsoPhalangeal-InterPhalangeal (MTP-IP) joint scales. The visual analog scale (VAS) , Short Form-12 Physical Component Score and Mental Component Score health summary scales (SF-12, PCS, MCS) were also used. Illustrated assessment was made with simple anteroposterior and profile radiographs of the foot, where hallux valgus angle (HVA) and intermetatarsal angle (IMA) were calculated. All the patients were examined and evaluated clinically. Radiographic assessment was performed preoperatively, 15 days, two months, 12 months and finally ~28 months postoperatively. All the pre- and post-operative outcomes were calculated with the use of the paired student’s t-test.

Methods

An innovative, alternative modified surgical technique was proposed for the treatment of the deformity, in moderate or even severe forms (HVA and IMA at 36° and 17°, respectively) with four steps. The first step includes the bunionectomy and secondly follows the osteotomy being performed with minimum tissue invasion and damage, protecting the vascularization of the distal bone (especially the head of the first metatarsal). The third step is the osteotomy being fixed with a cannulated screw for an extra stabilization, followed by careful release of the adductor hallucis distally of the lateral sesamoid bone after a good inspection intra-operatively, controlling the pulling strength of the tendon and the position of the sesamoid bone. The capsuloplasty (modified McBride capsuloplasty) is the fourth critical step of the surgery because it significantly improved the alignment of the first ray of the foot, and with an extended postoperative rehabilitation, it helped the prevention of the resurgence of this deformity. All patients were operated utilising a Chevron-type osteotomy with the use of compression and neutralization screws which are cannulated, self-drilling and self-taping. Their design allows them to distribute the compression effect and facilitate osteosynthesis. These screws are made of Titanium Alloy Ti6Al4V and are intended for the fixation of arthrodeses, osteotomies or fracture of long or short bones of the upper and lower limbs, as well as for osteosyntheses requiring a mono- or bi-cortical compression. 

Surgical technique

**Figure 1:**
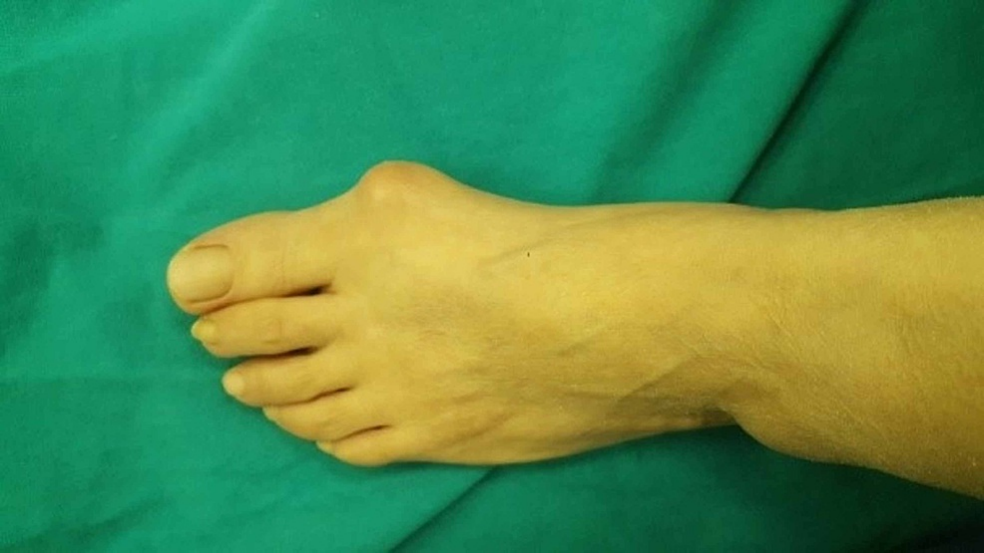
The deformity of the hallux valgus.

**Figure 2:**
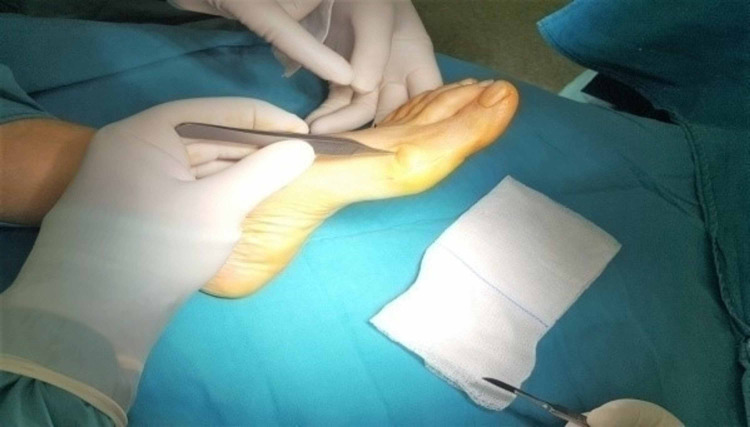
Medial longitudinal 3-4 cm skin incision.

**Figure 3:**
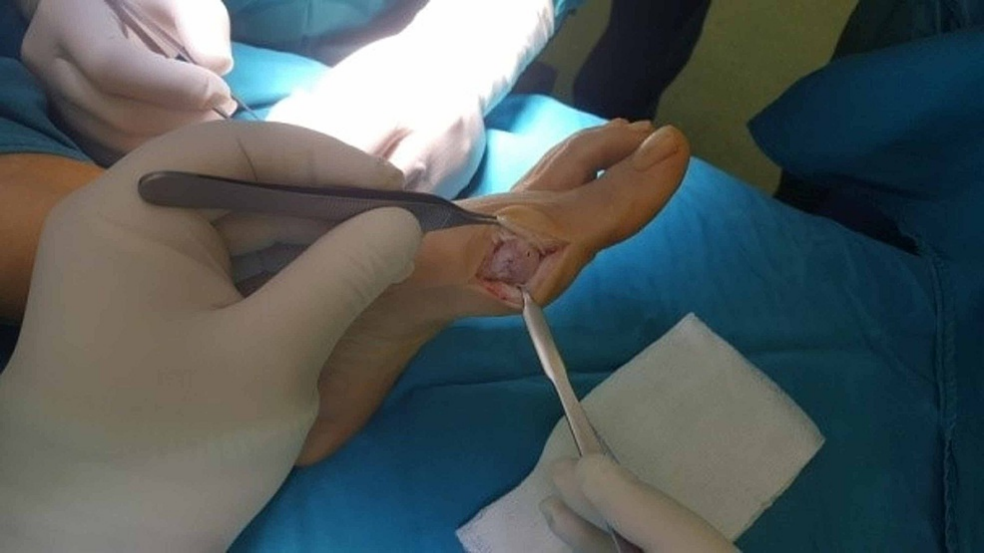
Longitudinal capsulotomy.

**Figure 4:**
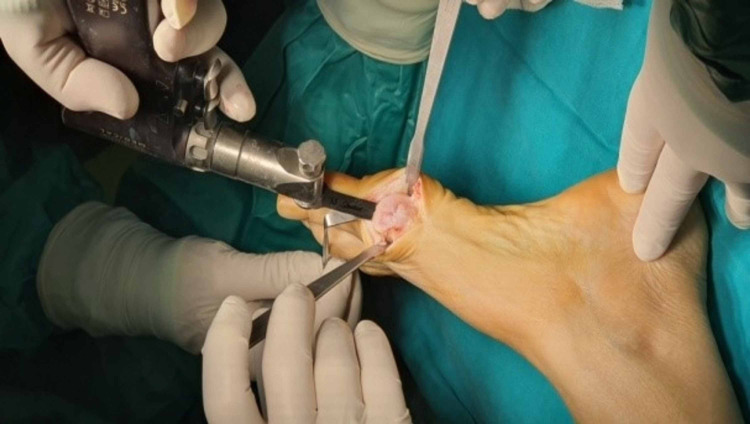
Overview of deformation and initial steps of surgical repair before “bunionectomy”.

**Figure 5:**
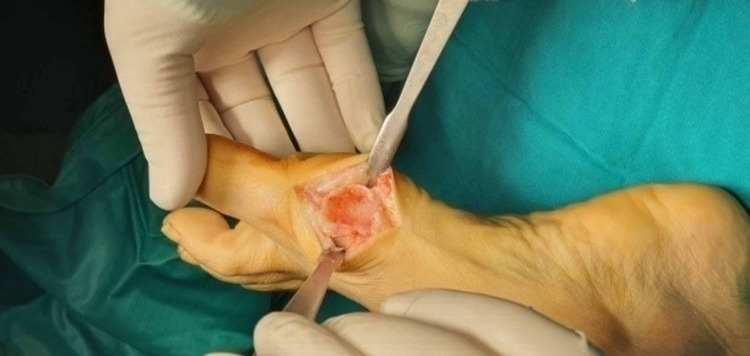
Overview after “bunionectomy”.

After a tourniquet is placed under the knee, a medial longitudinal skin incision of 3 cm to 4 cm was made, followed by a medial capsular incision proximal of the first phalanx and distal of the first metatarsal, identifying and protecting the dorsal and plantar vascular bundles. The distal end of the first MetaTarsoPhalangeal Joint (MTPJ) was dislocated with a mini Hohmann bone retractor (size 16/8) and the “Bunion” exostosis was cut longitudinally to the first metatarsal with a mini oscillating saw and eventually removed (Figures 1-5).

 

 

 

A second 12 mm incision was made, in first web space (between the first and second toe) for better visualization of the area laterally of the first metatarsal bone and the distal part of the adductor hallucis tendon was released from the lateral sesamoid bone and distally to the lateral proximal part (base) of the first phalanx of the first toe. The release was made just at the insertion of the tendon to the bone with a lancet (No11). The next step of soft-tissue rebalancing was the manual correction of the first toe to a varus position and the control of the tension of the deep transverse metatarsal ligament and the oblique head of the adductor hallucis tendon proximal to the lateral sesamoid with a curved Mayo scissor. Their release depended on the sesamoids and whether they were mobile and re-centered beneath the first metatarsal and generally the first ray of the first toe.

**Figure 6:**
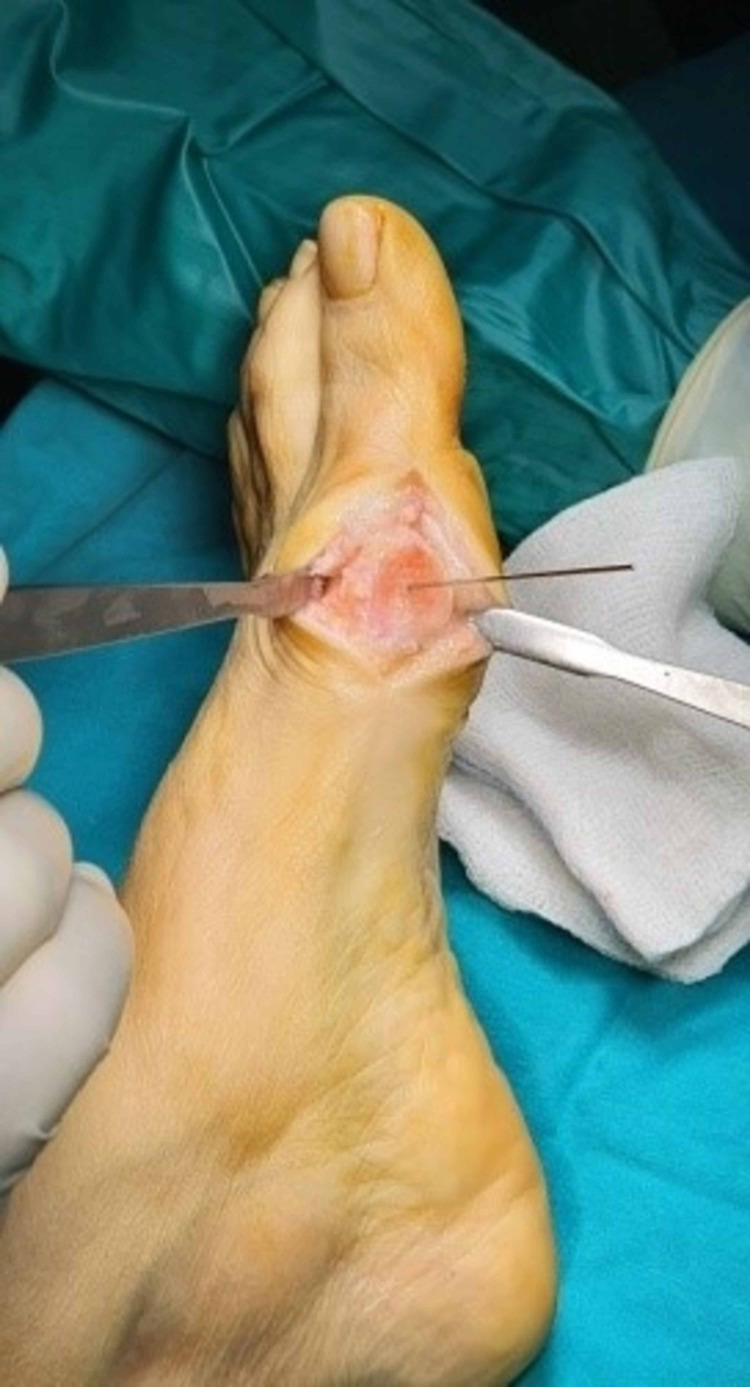
Marking the center of the first metatarsal head with a k-wire.

**Figure 7:**
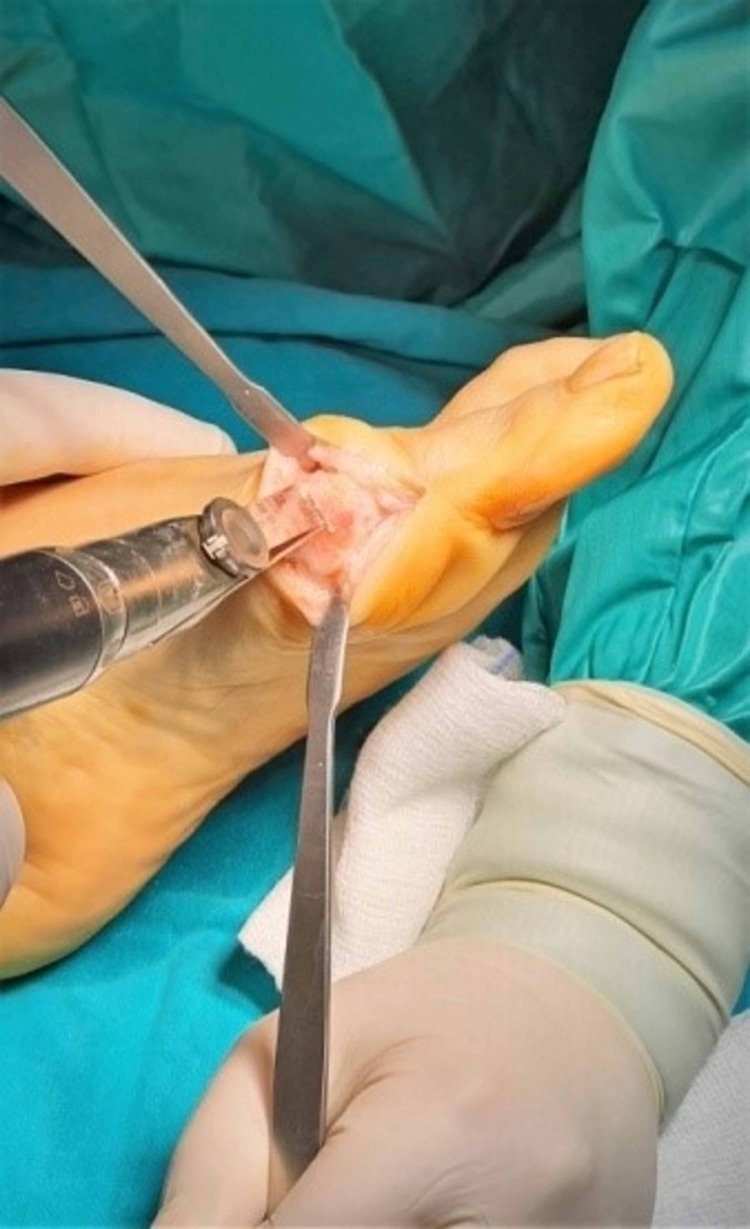
First-dorsal osteotomy.

**Figure 8:**
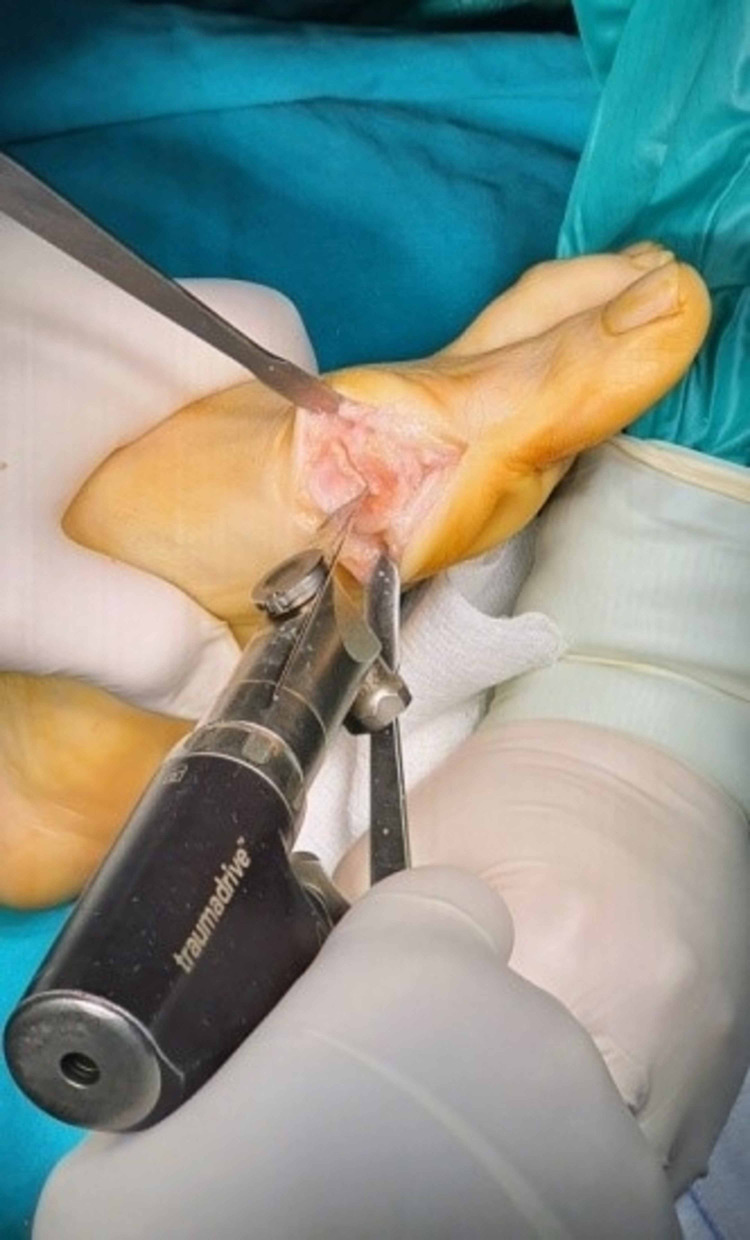
Second-plantar osteotomy

**Figure 9:**
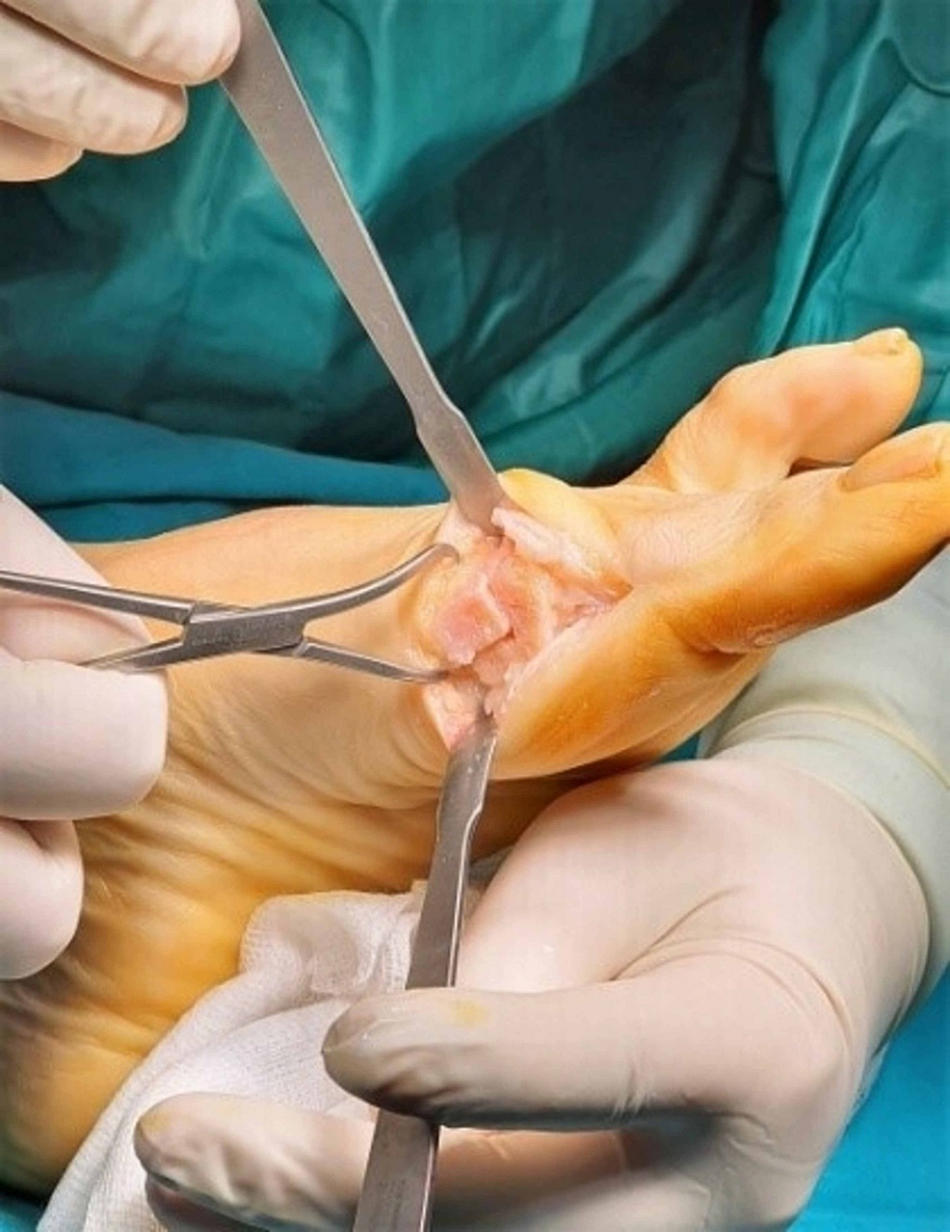
Translating the osteotomized head laterally with the help of a reduction forceps.

**Figure 10:**
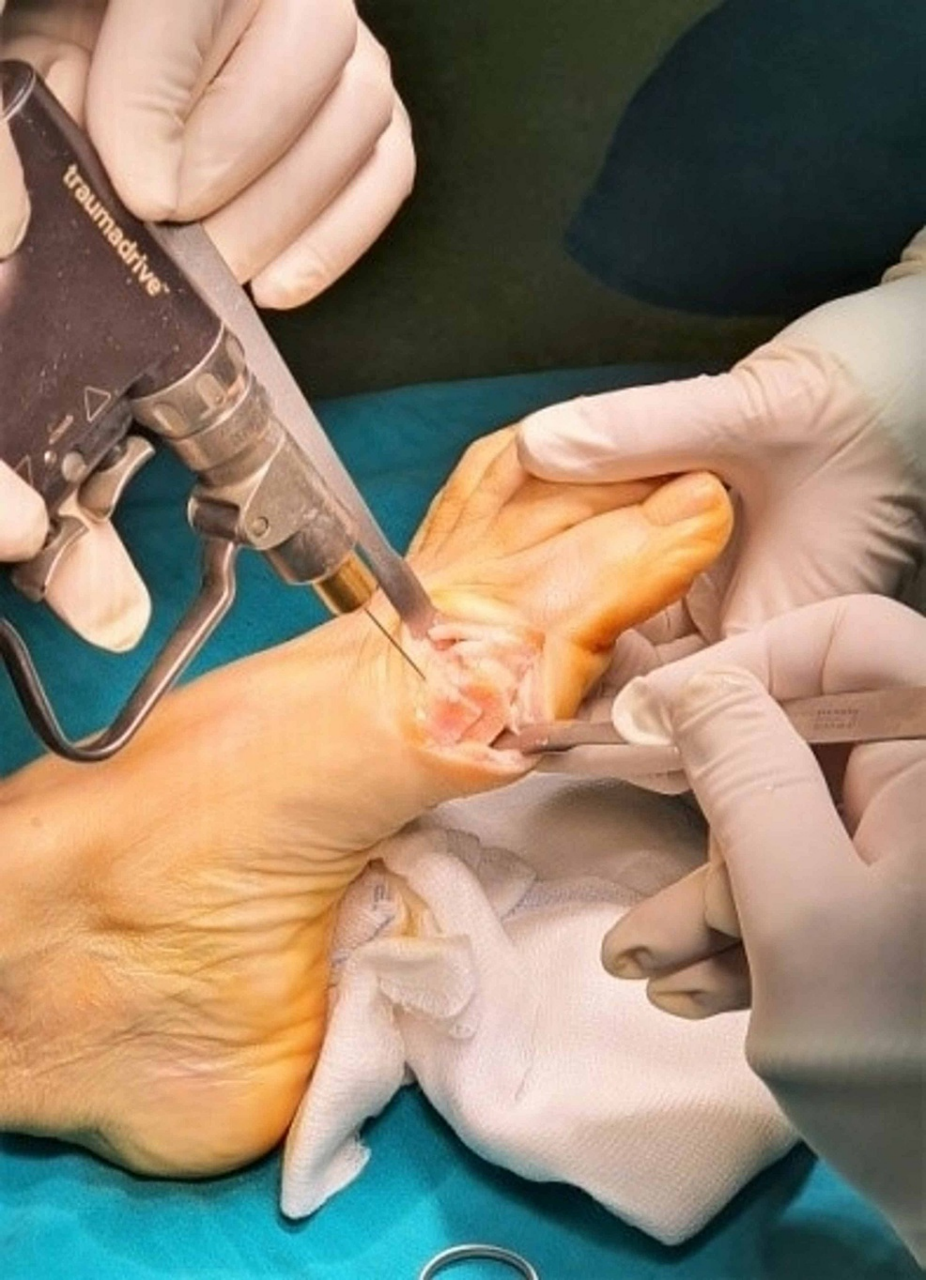
Temporal stabilization with a k-wire.

**Figure 11:**
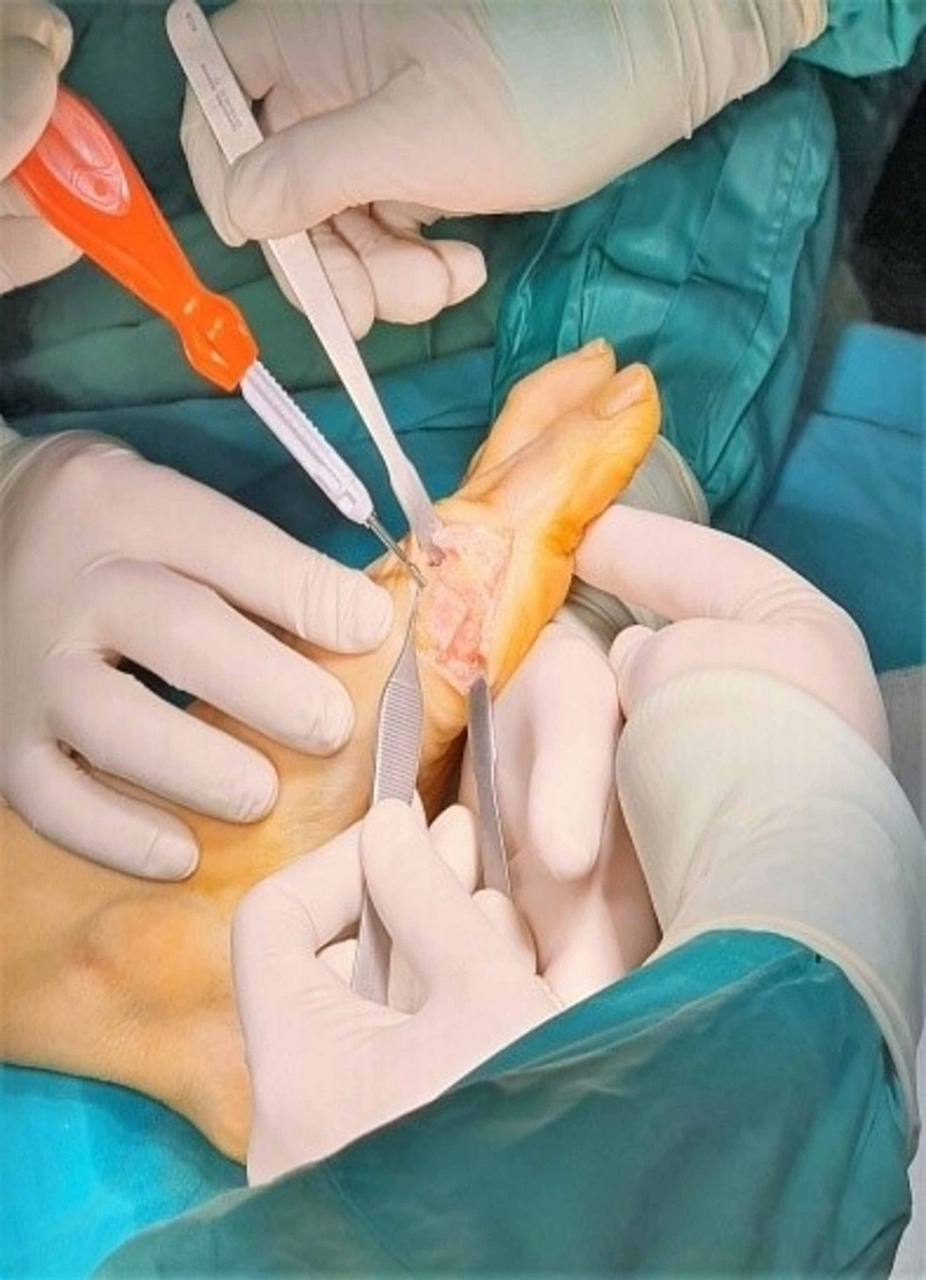
Measurement and screw installation.

**Figure 12:**
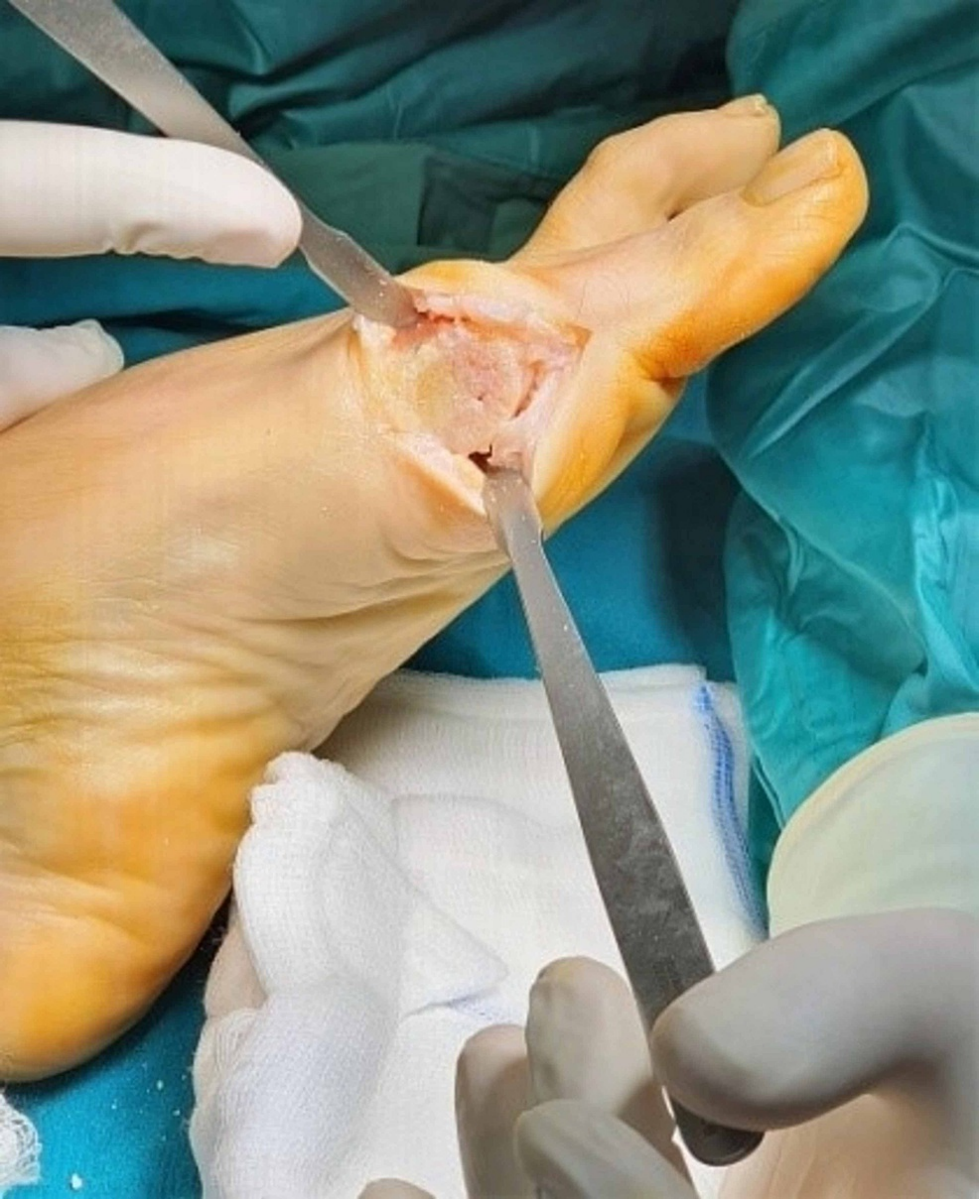
Remodeling of the osteotomy.

**Figure 13:**
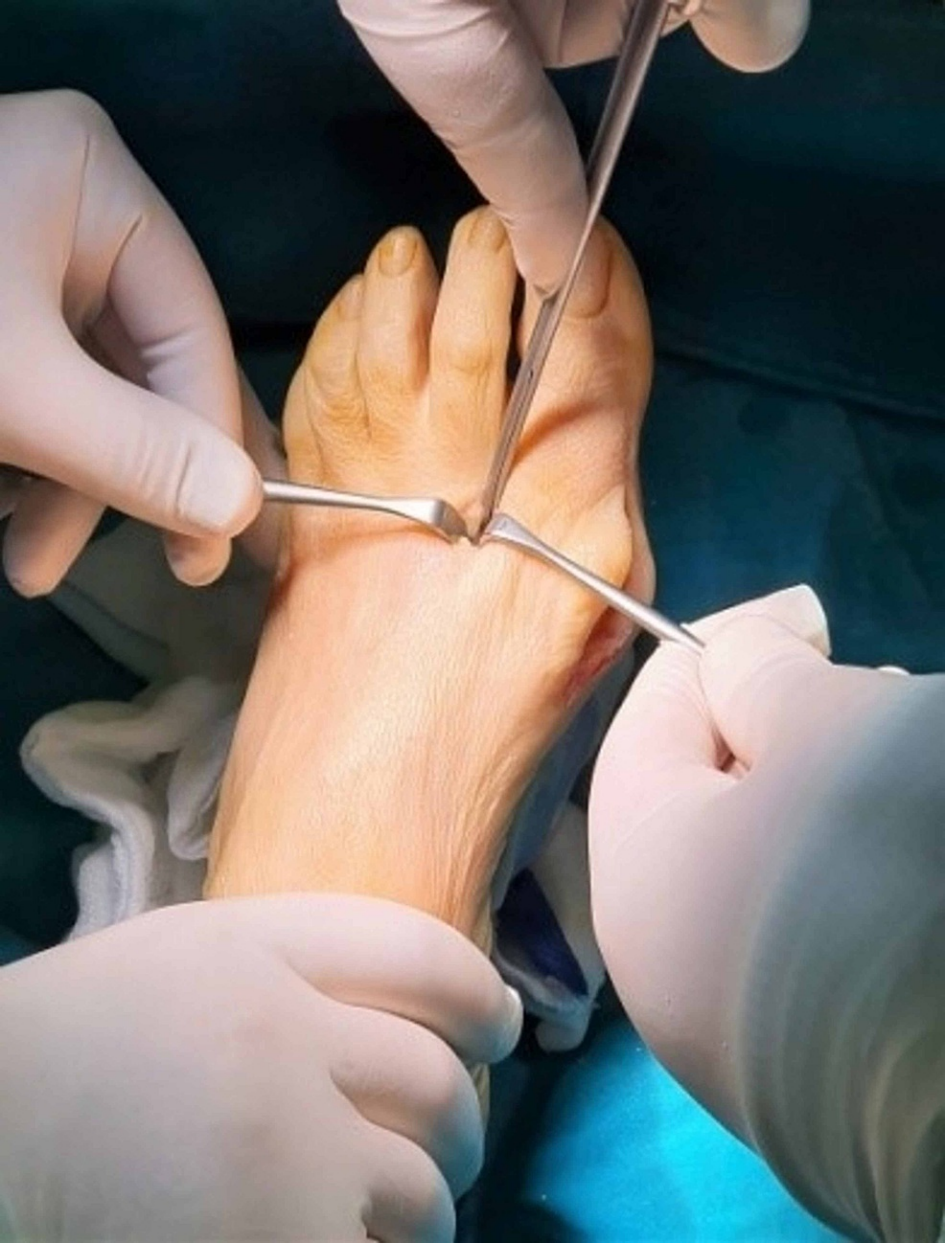
Lateral soft tissue release.

 The alternative modified Chevron osteotomy included firstly the mark with a k-wire in the center of the head of first metatarsal to direct correctly the dorsal as well as the plantar osteotomy. The dorsal osteotomy was always almost vertical inclination to the longitudinal axis of the first metatarsal (85° from the center of the metatarsal head dorsally just close to the articular cartilage). The second plantar osteotomy angle depended on the level of the deformity and it ranged from 100° to 80° according to the dorsal osteotomy. The greater the deformity, the closer to the horizontal axis of the first metatarsal bone the plantar osteotomy should be done for better displacement of the first metatarsal head laterally, preserving the intrinsic stability of the first metatarsophalangeal joint (MTPJ), depending on the degree of the deformity. When the IMA ranged from 11°-13° and the HVA from 19°-25° the cutting angle between the two osteotomies should be 100°, with the IMA from 13°- 16° and the HVA from 25°-33° the angle should be 90° and finally in severe forms of deformity with the IMA 16°-19° and the HVA 33°-42° the osteotomy angle should be at 80°, as close to the longitudinal axis of the bone as possible. The role of the K-wire was to move the head from the osteotomized bone until the detachment of them. Then the k-wire was removed and displaced the head laterally in an attempt to achieve the desirable axis of the first ray controlled with mini c -arm intensifier fluoroscopy. A second k-wire guide was used to stabilize the fragments with the help of a backhaus towel clip forceps to compress the gap of the fracture. The direction of the k-wire should always be vertical to the plantar osteotomy. Finally, after the measurement of the length of the aperture, a cannulated screw of 2.5 mm was inserted from a dorsal and proximal direction to a plantar and distal site of the osteotomy. Also, a preparation for the burial of the head of the screw in the proximal bone was done with a special instrument of the set (Figures 6-13).

**Figure 14:**
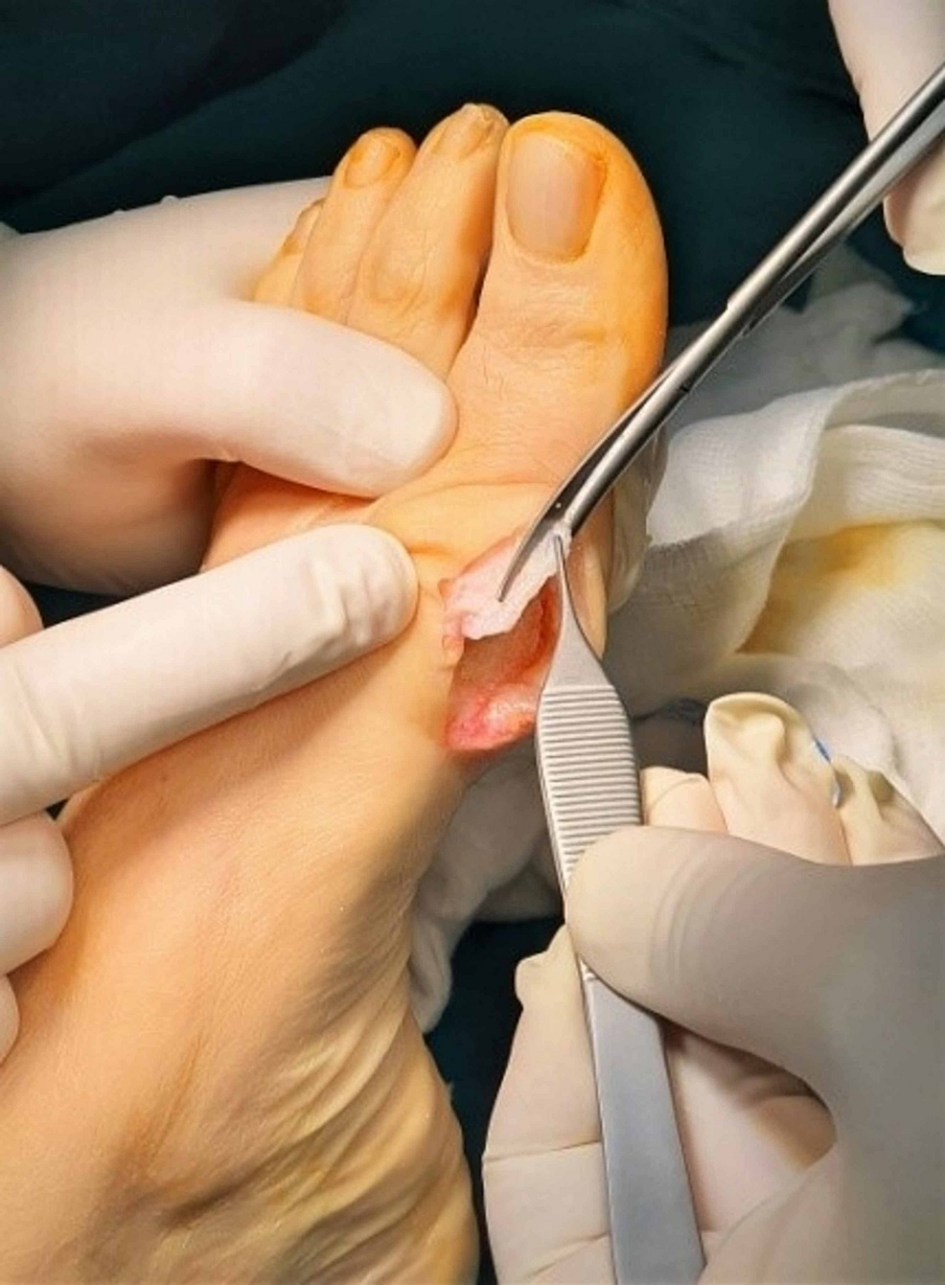
Excision of the excessive capsular tissue.

**Figure 15:**
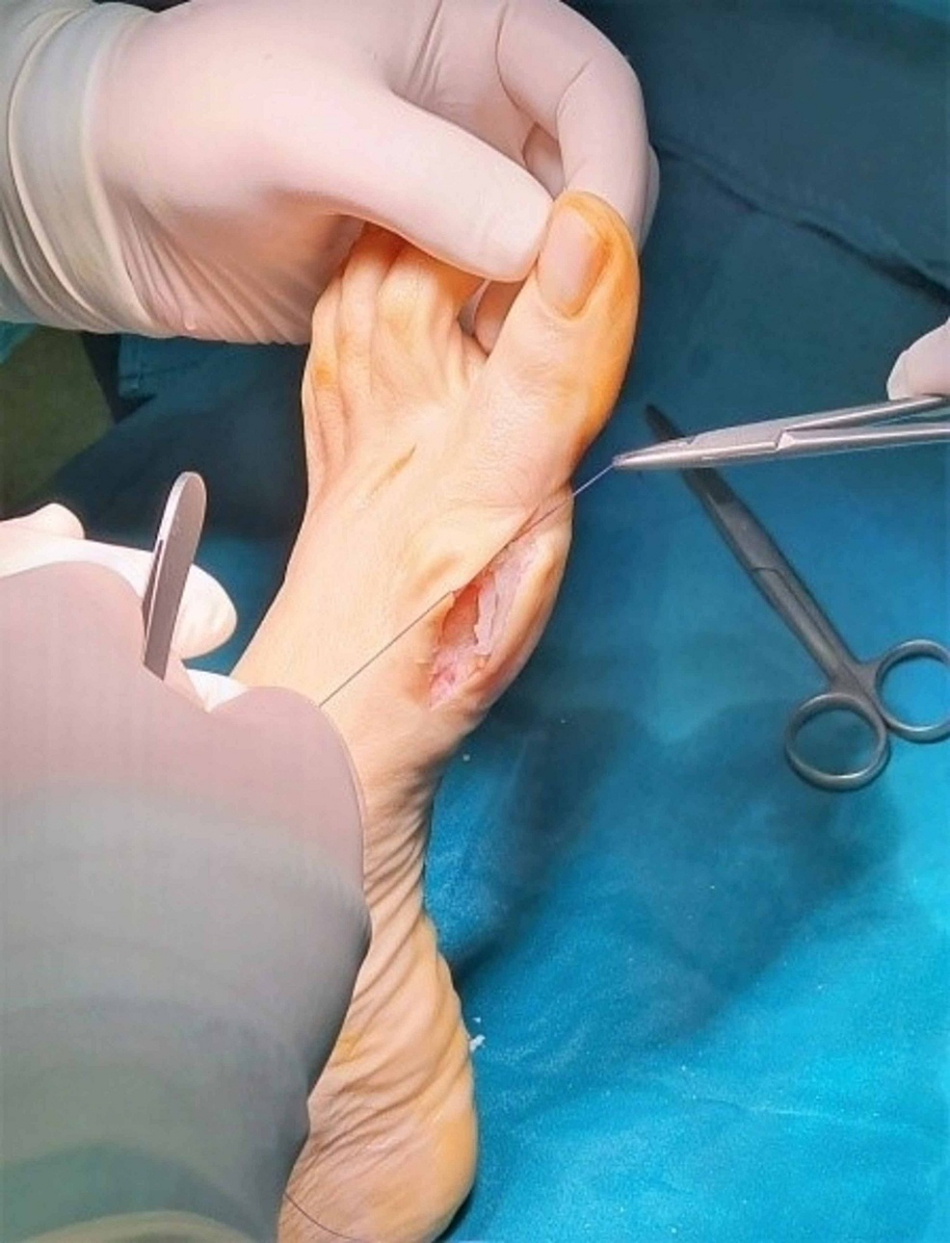
Capsulorrhaphy.

**Figure 16:**
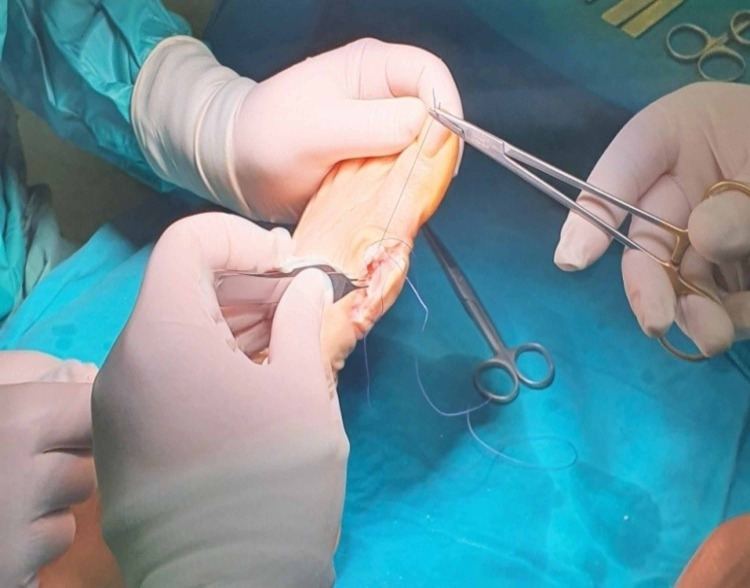
Subcuticular skin closure.

The capsuloplasty was a critical step in this surgical procedure because it defined and strengthened the correction of the alignment of the first foot ray. It is done with a continuous tight sewing with a strong absorbable suture, starting from and returning to the distal part of the capsule. The tightness of the sutures should depend on the preferred correction or overcorrection of the first foot-ray alignment. Finally, the remodeling of the remaining margins of the capsule and the reformation of the skin was mandatory (Figures 14-16).

Postoperative care

The postoperative care played a vital role and consists of two periods. The first postoperative care period lasted about six to seven weeks and the second postoperative conservative care two to six months. A tension functional bandage was applied immediately after the surgery with the first toe in overcorrection until the second wound dressing change three days later, with weekly changes, maintaining the appropriate direction of the first foot ray until the final removal of the bandages six to seven weeks after surgery. The patient stayed in the hospital for a few hours where he was treated with antibiotics and anticoagulant of low molecular weight. Full weight-bearing was allowed from the first day with the patient wearing a special shoe for about two months, during which radiograph was used to assess the ossification of the osteotomies. The second care period included the maintenance of the first toe alignment with the application of a soft pad between the first and second toes and the wearing of soft and special wide shoes.

## Results

Clinical outcomes

All the clinical scores were improved significantly, as the average value of the AOFAS-MTP-IP score increased from 47.2 points preoperatively (range 44-55) to a final 92.4 points postoperatively (range 85-94) with p≤0.028, whereas the average value of SF-12 score PCS/MCS was 33.4/34 points preoperatively (range 31-35/31.8-35) and 53/58.7 points postoperatively (range 50-54/57.5-59), with p≤0.032 and p≤0.02, respectively, and the average VAS score decreased from 7.2 points preoperatively (range 6-8) to 1.1 points postoperatively (range 0-1.5) with p≤0.041.

Radiological findings

**Figure 17:**
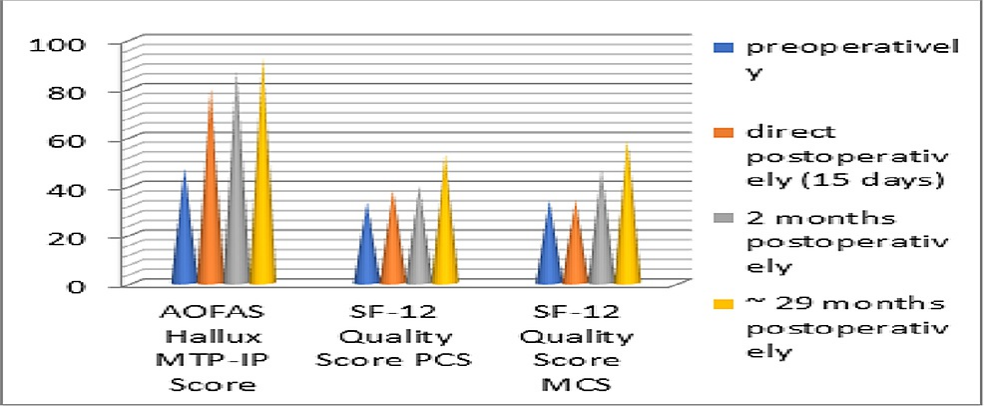
Results of the AOFAS Hallux MTP-IP and SF-12 PCS-MCS scores. AOFAS: American Orthopedic Foot and Ankle Society; MTP-IP: MetaTarsoPhalangeal-InterPhalangeal; PCS: physical component score; MCS: mental component score; SF-12: Short Form-12.

**Figure 18:**
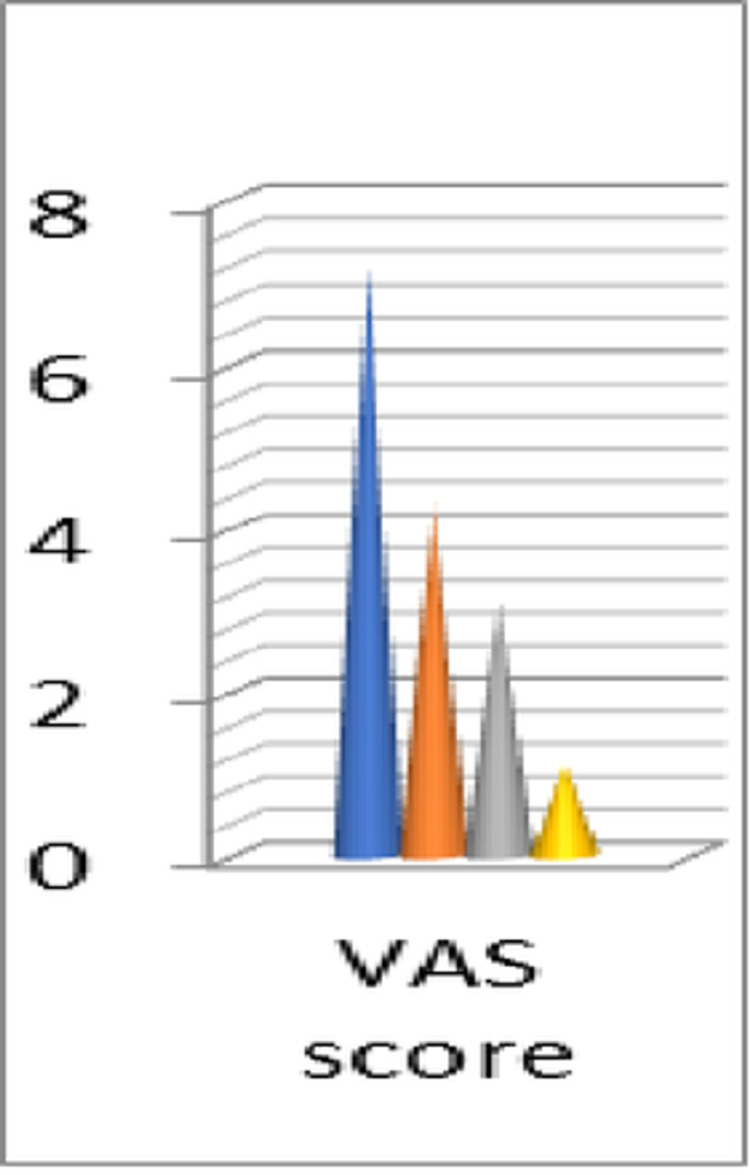
Results of the VAS score. VAS: visual analog scale.

**Figure 19:**
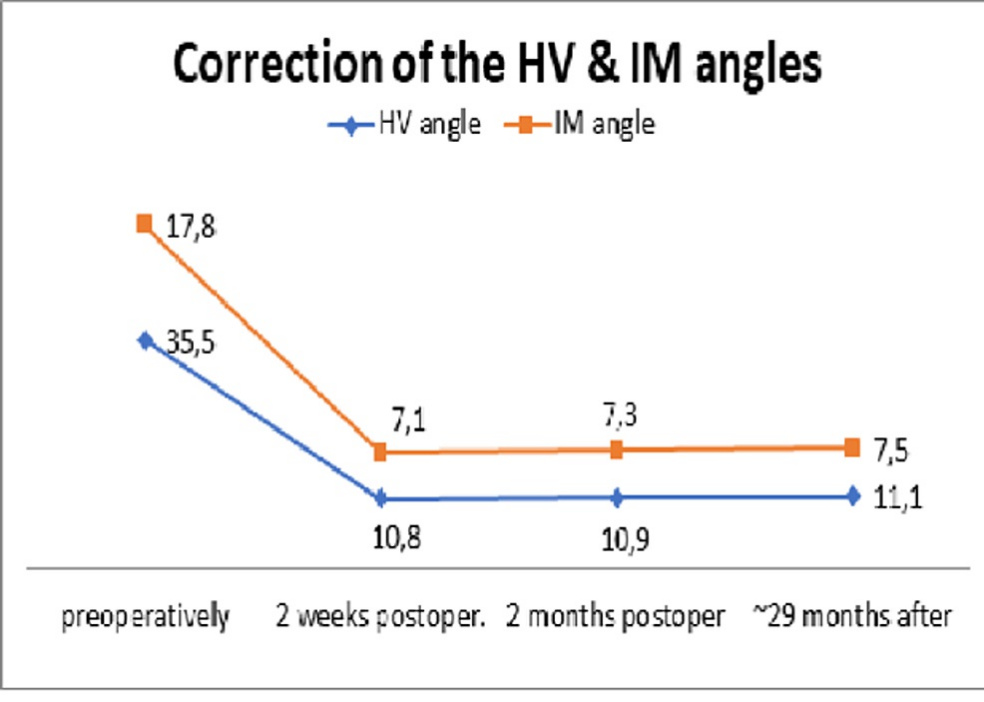
Diagram of correction of the HVA and IMA. HVA: hallux valgus angle; IMA: intermetatarsal angle.

Regarding the radiological findings, the average value of HVA and IMA decreased and remained consistent with the initial post-operational correction angles for 2 1/2 years after the last follow-up. The average pre-operative HVA decreased from 35.5° (range 29-37.6°) to 11.1° post-operatively (range 8.8°-12.3°) in the last follow-up (p ≤ 0.034) and the average IMA from17.8° preoperatively (range 15.1°-21.8°) to 7.5° post-operatively (range 6.3°-9.6°) in last follow up (p ≤ 0.038). The following table describes in detail all the scores and angles of the cohort study (Table 1) with charts depicting the clinical scores (Figures 17, 18) as well as the correction angles (Figure 19).

**Table 1:** Clinical outcome scores. VAS: visual analog scale; AOFAS: American Orthopedic Foot and Ankle Society; MTP-IP: MetaTarsoPhalangeal-InterPhalangeal; PCS: physical component score; MCS: mental component score; SF-12: Short Form-12: HVA: hallux valgus angle; IMA: intermetatarsal angle.

	Preoperatively	Final follow-up ~29 months	p-value
Mean AOFAS MTP-IP score	47.2 ± 7.8	92.4 ± 7.4	0.028
Mean SF-12 Quality score PCS	33.4 ± 2.4	53 ± 3	0.032
Mean SF-12 Quality score MCS	34 ± 2.2	58.7 ± 1.2	0.026
Mean VAS score	7.2 ± 1	1.1 ± 1	0.041
Mean HVA	35.5^ο^ ± 6.5^ο^	11.1^ο^ ± 2.3^ο^	0.034
Mean IMA	17.8^ο^ ± 4^ο^	7.5^ο^ ± 2.1^ο^	0.038

 

**Figure 20:**
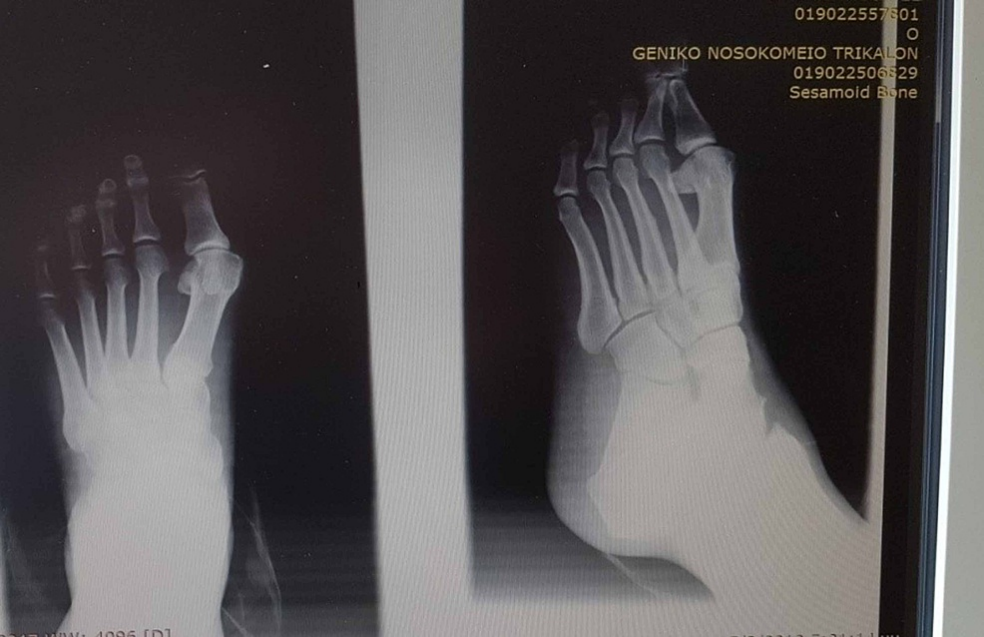
Man - 47-year-old medic (GP) with moderate deformity preoperatively.

**Figure 21:**
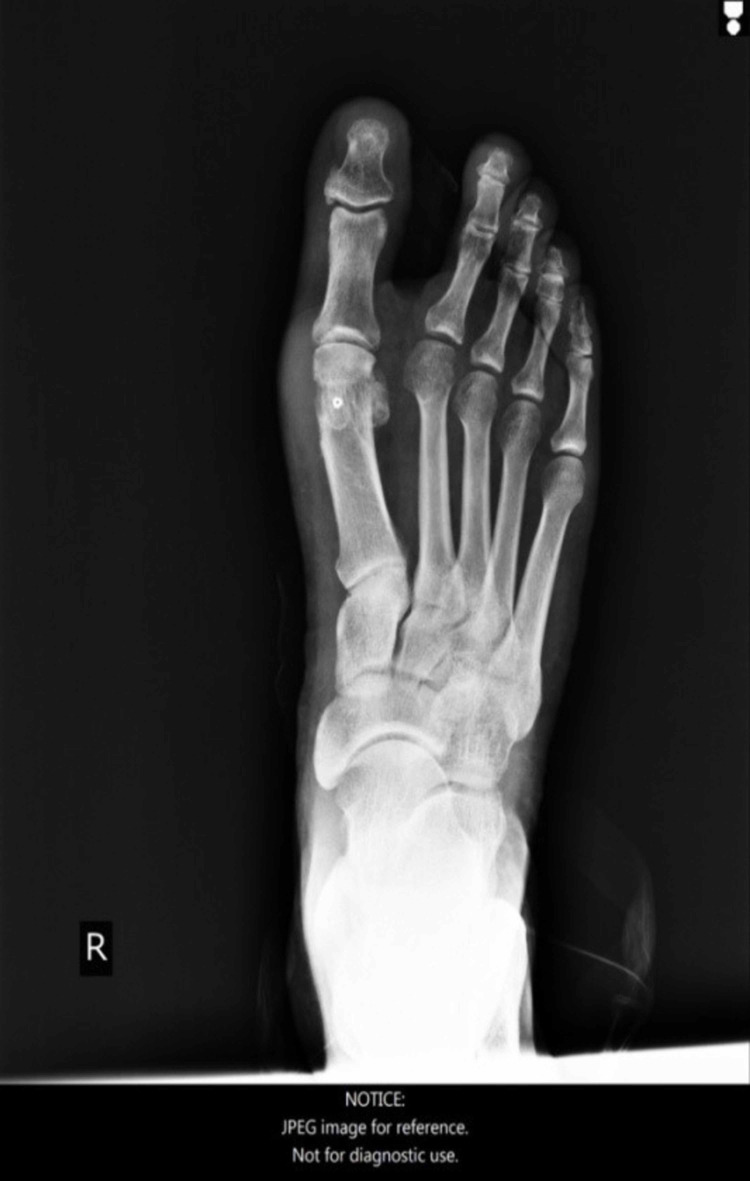
Foot X-ray, anteroposterior view, 27 months post-operatively.

**Figure 22:**
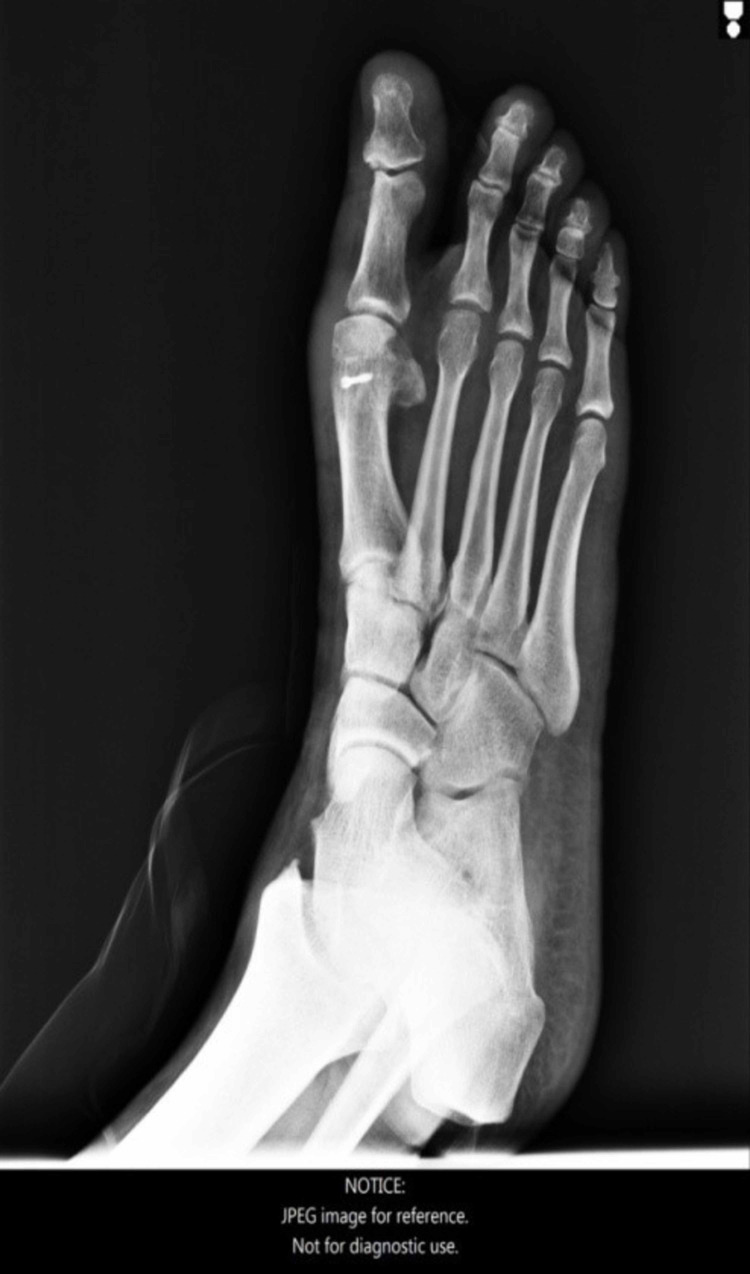
Foot X-ray, lateral view, 27 months post-operatively.

**Figure 23:**
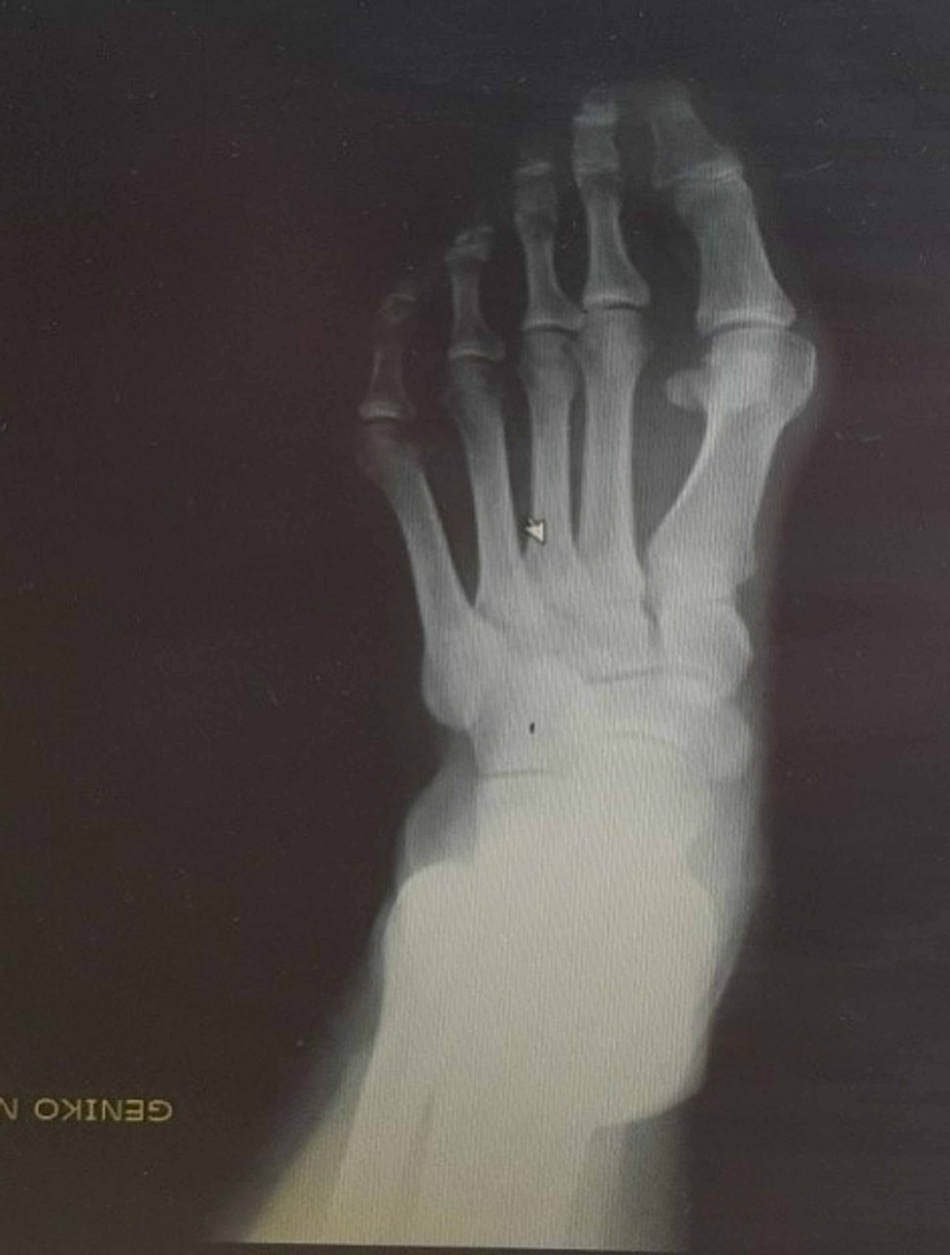
Woman - 52-year-old housewife with moderate deformity preoperatively.

**Figure 24:**
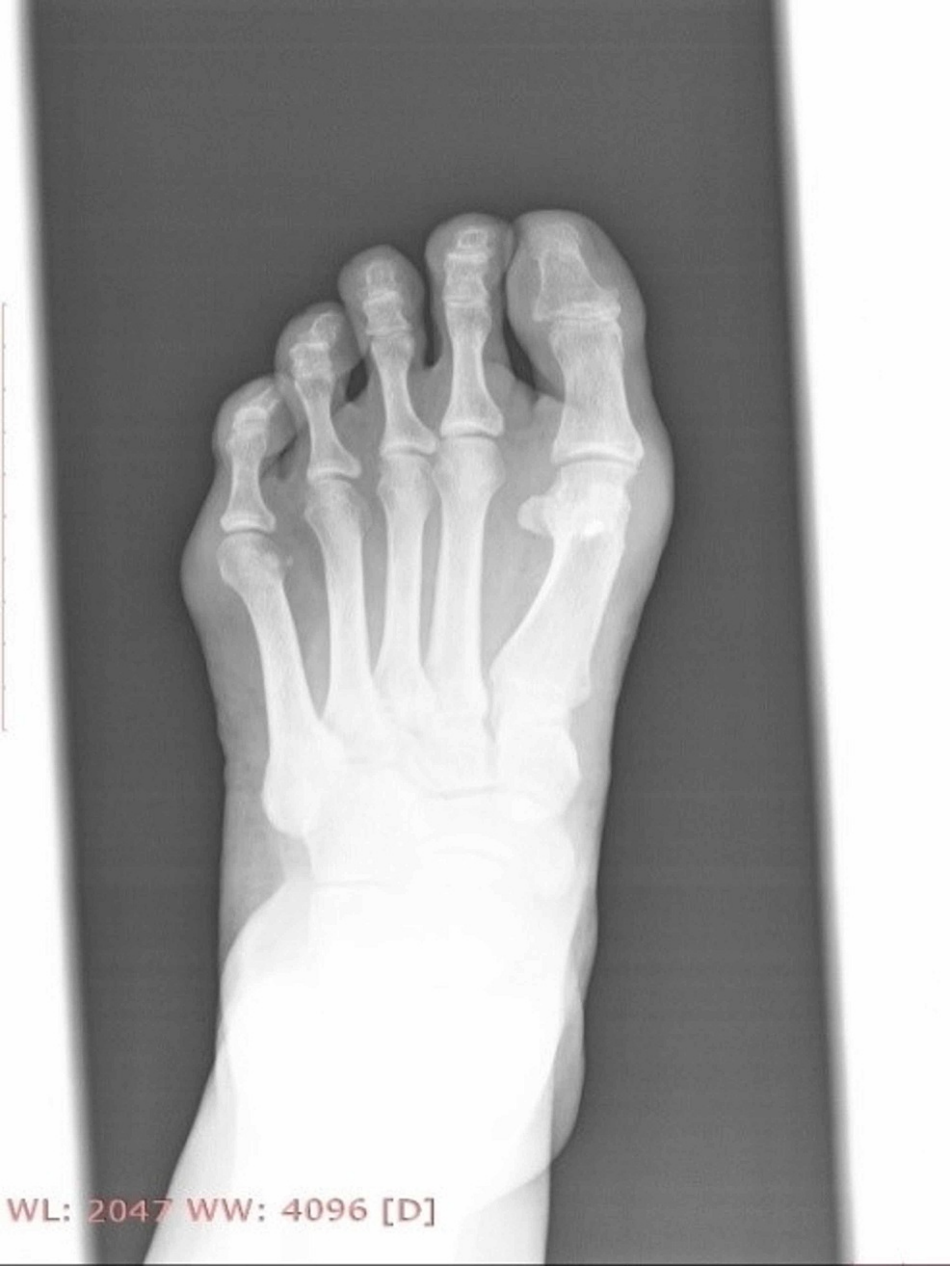
Clinical picture of the foot after 29 months post-operatively.

All osteotomies healed within four to six weeks after surgery. None of the patients experienced pain directly after the operation or later and none of the patients experienced any fracture or cut out of the osteosynthesis hardware. No infections were observed. Additionally, no cases of delayed union or nonunion of the osteotomies, no necrosis of metatarsal head, no stiffness of the first metatarsophalangeal joint or no Sudeck syndrome were observed. Until the last follow-up, all 36 patients were completely satisfied clinically and aesthetically with this surgical treatment and the survival rate in this mid-term follow-up cohort study was 100%. Figures 20-24 show some radiological and clinical outcomes preoperatively and in the last follow-up. There was no loss of follow-up regarding any patient taking part in this study.

## Discussion

The surgical difficulties and the decision for the osteotomy to treat HV are known because of the special character and the biomechanical specificities of this part of the foot [2]. A few years ago, the Chevron osteotomy was a well-established technique for the correction of mild to moderate deformities of HV [3,4]. Later, introducing a combination of other surgical procedures, such as Akin osteotomy and better soft tissue release, many studies have proved that the Chevron osteotomy could allow corrections of moderate to severe deformities as well [5,6].

Many discussions are taking place about the different types of the moderate Chevron osteotomies or other multiplanar osteotomies of the first metatarsal, which lead to an adequate correction of the deformity, avoiding the resurgence of hallux valgus or the development of arthritis, metatarsalgia leading inevitably to revision [7]. Nevertheless, there are obstacles and many complications [8] associated with the combined or multiplanar osteotomies, such as osteonecrosis of the metatarsal head, non-unions or malunions, infections after large detachments, blind oblique or horizontal cut orientation which disturbs this foot area with these demanding techniques, proving once again the ineffectiveness and the difficulty in deciding on an effective method [9,10].

An alternative modified Chevron osteotomy [11], as described above, focused easily and with respect to the surrounding soft tissues to achieve the elimination of the deformity, preserving the natural architecture of a healthy foot with all the stabilizing intrinsic factors, and the congruity of the MTPJ remaining stable. Maintaining this way and protecting the vascular supply of the distal bone provided safety for the union of the bone, whereas the insertion of a cannulated screw, like a lag screw, just vertical to the osteotomized line, ensured the impaction of the cancellous bone of the head to the shaft of the metatarsal. Therefore, this alternative modified Chevron osteotomy seemed quite promising for the successful correction of larger to moderate deformities in combination with the right and focused soft tissue release and the strength of the capsulorrhaphy-capsuloplasty [12]. Finally, the postoperative care and its duration played a major role because if there are no disturbances of the intrinsic factors due to the shape of osteotomy, the patient had the ability to walk immediately after surgery, which also helped the healing of the fracture. Additionally, the cost of a single screw was quite low.

A less invasive Chevron osteotomy, in combination with modified McBride capsuloplasty [5], with an emphasis on proper respect for the anatomy of the region, as well as the very careful and appropriate medical care from the first day postoperatively and the patient’s personal care of their foot for as long as possible provided very satisfactory results even in heavy Hallux Valgus cases with half a day hospitalization.

Limitations

Limitations of the study included the relatively small number of patients and the lack of a control group. The small number of patients prevented the statistical analysis for finding risk factors that could contribute to the failure of the treatment.

## Conclusions

This alternative moderate Chevron osteotomy and modified McBride capsuloplasty was an effective method for the treatment of hallux valgus in patients over 55 years old. Clinical and radiologic results were satisfactory and the survival rate is 100%, ~ 2 1/2 years after surgery. Proper postoperative recovery and the maintenance and protection of soft tissues with special soft inserts maintained and improved the subsequent course of operated hallux valgus.
